# EANO guidelines on the diagnosis and treatment of diffuse gliomas of adulthood

**DOI:** 10.1038/s41571-020-00447-z

**Published:** 2020-12-08

**Authors:** Michael Weller, Martin van den Bent, Matthias Preusser, Emilie Le Rhun, Jörg C. Tonn, Giuseppe Minniti, Martin Bendszus, Carmen Balana, Olivier Chinot, Linda Dirven, Pim French, Monika E. Hegi, Asgeir S. Jakola, Michael Platten, Patrick Roth, Roberta Rudà, Susan Short, Marion Smits, Martin J. B. Taphoorn, Andreas von Deimling, Manfred Westphal, Riccardo Soffietti, Guido Reifenberger, Wolfgang Wick

**Affiliations:** 1 Department of Neurology, Clinical Neuroscience Center, University Hospital and University of Zurich, Zurich, Switzerland; 2 Brain Tumor Center at Erasmus MC Cancer Institute, University Medical Center Rotterdam, Rotterdam, Netherlands; 3 Division of Oncology, Department of Medicine I, Medical University of Vienna, Vienna, Austria; 4 Department of Neurosurgery, Clinical Neuroscience Center, University Hospital and University of Zurich, Zurich, Switzerland; 5 University of Lille, U1192 Lille, France; 6 Centre Hospitalier Universitaire (CHU) Lille, Neuro-Oncology, General and Stereotaxic Neurosurgery Service, Lille, France; 7 Oscar Lambret Center, Neurology, Lille, France; 8 Department of Neurosurgery, University Hospital Munich LMU, Munich, Germany; 9 Radiation Oncology Unit, Department of Medicine, Surgery and Neurosciences, University of Siena, Siena, Italy; 10 Department of Neuroradiology, University Hospital Heidelberg, Heidelberg, Germany; 11 Catalan Institute of Oncology (ICO), Hospital Germans Trias i Pujol, Badalona, Spain; 12 Aix-Marseille Université, Assistance Publique–Hôpitaux de Marseille (APHM), CHU Timone, Department of Neuro-Oncology, Marseille, France; 13 Department of Neurology, Leiden University Medical Center, Leiden, Netherlands; 14 Department of Neurology, Haaglanden Medical Center, The Hague, Netherlands; 15 Department of Neurology, Erasmus MC, Rotterdam, Netherlands; 16 Department of Clinical Neurosciences, University Hospital Lausanne, Lausanne, Switzerland; 17 Department of Neurosurgery, Sahlgrenska University Hospital, Gothenburg, Sweden; 18 Institute of Neuroscience and Physiology, Department of Clinical Neuroscience, Sahlgrenska Academy, Gothenburg, Sweden; 19 Department of Neurology, Medical Faculty Mannheim, Mannheim Center for Translational Neuroscience (MCTN), Heidelberg University, Mannheim, Germany; 20 German Consortium of Translational Cancer Research (DKTK), Clinical Cooperation Unit Neuroimmunology and Brain Tumor Immunology, German Cancer Research Center (DKFZ), Heidelberg, Germany; 21 Department of Neuro-Oncology, University Hospital, Turin, Italy; 22 Leeds Institute of Medical Research, St James’s University Hospital, Leeds, UK; 23 Department of Radiology and Nuclear Medicine, Erasmus MC, University Medical Center Rotterdam, Rotterdam, Netherlands; 24 Department for Neuropathology, University Hospital Heidelberg, Heidelberg, Germany; 25 DKTK and Clinical Cooperation Unit Neuropathology, DKFZ, Heidelberg, Germany; 26 Department of Neurosurgery, University Hospital Hamburg, Hamburg, Germany; 27 Department of Neuropathology, Heinrich Heine University Düsseldorf, Düsseldorf, Germany; 28 DKTK partner site Essen/Düsseldorf, Düsseldorf, Germany; 29 Neurology Clinic and National Center for Tumor Diseases, University Hospital Heidelberg, Heidelberg, Germany; 30 DKTK and Clinical Cooperation Unit Neurooncology, DKFZ, Heidelberg, Germany

**Keywords:** CNS cancer, CNS cancer

## Abstract

In response to major changes in diagnostic algorithms and the publication of mature results from various large clinical trials, the European Association of Neuro-Oncology (EANO) recognized the need to provide updated guidelines for the diagnosis and management of adult patients with diffuse gliomas. Through these evidence-based guidelines, a task force of EANO provides recommendations for the diagnosis, treatment and follow-up of adult patients with diffuse gliomas. The diagnostic component is based on the 2016 update of the WHO Classification of Tumors of the Central Nervous System and the subsequent recommendations of the Consortium to Inform Molecular and Practical Approaches to CNS Tumour Taxonomy — Not Officially WHO (cIMPACT-NOW). With regard to therapy, we formulated recommendations based on the results from the latest practice-changing clinical trials and also provide guidance for neuropathological and neuroradiological assessment. In these guidelines, we define the role of the major treatment modalities of surgery, radiotherapy and systemic pharmacotherapy, covering current advances and cognizant that unnecessary interventions and expenses should be avoided. This document is intended to be a source of reference for professionals involved in the management of adult patients with diffuse gliomas, for patients and caregivers, and for health-care providers.

## Abstract (web summary)

Herein, the European Association of Neuro-Oncology (EANO) provides recommendations for the diagnosis, treatment and follow-up of adult patients with diffuse gliomas. These evidence-based guidelines incorporate major changes in diagnostic algorithms based on the 2016 update of the WHO Classification of Tumors of the Central Nervous System as well as on evidence from recent large clinical trials.

## Introduction

The classification of gliomas has undergone major changes through the revision of the fourth edition of the WHO Classification of Tumors of the Central Nervous System^1^ in 2016. Further refinements of the classification were subsequently proposed by the Consortium to Inform Molecular and Practical Approaches to CNS Tumour Taxonomy — Not Officially WHO (cIMPACT-NOW)^2–4^. These documents enable a diagnosis of glioblastoma to be made not only based on histology but also on the basis of several molecular markers and propose the discontinuation of the term ‘IDH-mutant glioblastoma’. To reflect these changes, the European Association of Neuro-Oncology (EANO) considered it necessary to update its guidelines for the management of adult patients with gliomas^5^ (Box 1). In the present evidence-based guidelines, we cover the prevention, early diagnosis and screening, integrated histomolecular diagnostics, therapy and follow-up monitoring of adult patients with diffuse gliomas. Aspects such as differential diagnosis, adverse effects of treatment, and supportive and palliative care are beyond the scope of this guideline document.

Box 1 Key new developments in the diagnosis and management of gliomas (2016–2020)

- Glioblastoma is now defined as a diffuse astrocytic glioma with no mutations in IDH genes nor histone H3 genes and is characterized by microvascular proliferation, necrosis and/or specific molecular features, including *TERT* promoter mutation, *EGFR* gene amplification and/or a +7/–10 cytogenetic signature.
- IDH-mutant glioblastoma is now referred to as IDH-mutant astrocytoma, WHO grade 4.
- Homozygous deletion of *CDKN2A/B* locus is a molecular marker of WHO grade 4 in IDH-mutant astrocytomas.
- Histone H3.3 G34-mutant diffuse hemispheric gliomas constitute a novel glioma entity corresponding to WHO grade 4.
- The value of the distinction between WHO grades 2 and 3 in IDH-mutant gliomas is increasingly challenged, and ongoing clinical trials (such as CODEL^83^ and EORTC 1635 (ref.^125^)) are enrolling patients with tumours of both grades.
- In the CATNON trial^89^, the combination of maintenance temozolomide with radiotherapy prolonged survival only in patients with IDH-mutant gliomas of WHO grade 3 and not in those with tumours diagnosed as IDH-wild-type anaplastic gliomas.
- The prolongation of maintenance temozolomide from 6 to 12 cycles extends neither progression-free survival nor overall survival^106^.
- Bevacizumab does not prolong progression-free survival nor overall survival in patients with 1p/19q-intact recurrent WHO grade 2 or 3 glioma^14^.
- Nivolumab is not superior to bevacizumab in patients with recurrent glioblastoma^119^.
- Nivolumab is not superior to temozolomide in patients with newly diagnosed glioblastoma without *MGMT* promoter methylation^100^.

## Methods

These evidence-based guidelines were formulated by a task force nominated by the EANO Executive Board following a proposal by the Chair of the EANO guidelines committee. This task force includes representatives of all the disciplines involved in the diagnosis and care of adults with glioma and reflects the multinational character of EANO. References were retrieved from the PubMed database using the search terms ‘glioma’, ‘anaplastic’, ‘astrocytoma’, ‘oligodendroglioma’, ‘glioblastoma’, ‘trial’, ‘clinical’, ‘surgery’, ‘radiotherapy’ and ‘chemotherapy’ between January 2011 and July 2020. Publications were also identified through searches of the authors’ own libraries. Only publications in English were reviewed. Data available only in abstract form were included in exceptional circumstances. The definitive reference list was generated based on relevance to the broad scope of these guidelines. The consensus recommendations were achieved through repeated circulation of manuscript drafts and telephone conferences involving members of the task force to discuss the most controversial areas. The key recommendations for the diagnosis and management of diffuse gliomas of adulthood, with their class of evidence (C) and level of recommendation (L)^6^ are reported at the end of each corresponding paragraph.

## Epidemiology and prevention

The annual incidence of gliomas is approximately of six cases per 100,000 individuals worldwide. Men are 1.6-fold more likely to be diagnosed with gliomas than women^7^. While the vast majority of cases are sporadic, certain familial tumour syndromes are associated with gliomagenesis, including neurofibromatosis type I, tuberous sclerosis, Turcot syndrome, Li–Fraumeni syndrome and Lynch syndrome. Screening with neuroimaging is limited to patients with such syndromes at the initial diagnostic work-up^8^. Repeat neuroimaging is not indicated unless new neurological symptoms and signs, such a seizures, aphasia, hemiparesis or sensory deficits, develop that suggest an intracranial lesion. The counselling and screening of asymptomatic relatives of patients with glioma who are found to be carriers of germline mutations associated with gliomagenesis should be conducted with caution and in cooperation with clinical geneticists. No known measures to prevent the development of gliomas exist.

## History and clinical examination

The evolution of neurological symptoms and signs enables the estimation of the growth dynamics of gliomas: tumours that cause symptoms only weeks before diagnosis are usually fast growing whereas those that cause symptoms for years before being diagnosed are usually slow growing. In most individuals, the symptoms and signs reported the year before diagnosis are non-specific (for example, fatigue or headache)^9–11^. A discussion of the patient’s history might reveal familial risk or rare exogenous risk factors (such as exposure to radiation) associated with the development of brain tumours. Information from relatives might be required to obtain a reliable history. Firm recommendations on when and how to involve family members and caregivers and how to assess the medical decision-making capacity in patients with brain tumours remain to be developed^12^.

Characteristic modes of clinical presentation include new-onset epilepsy, focal deficits (such as pareses or sensory disturbances), neurocognitive impairment, and symptoms and signs of increased intracranial pressure. The physical examination of patients with brain tumours focuses on the detection of systemic cancer to differentiate primary brain tumours from brain metastases and contraindications for neurosurgical procedures. The Neurological Assessment in Neuro-Oncology (NANO) scale can be used to document some of the results of the neurological examination^13^. Neurocognitive assessment using a standardized test battery^14^, beyond documenting performance status and performing a Mini Mental State Examination (MMSE)^15^ or a Montreal Cognitive Assessment (MoCA)^16^, has become increasingly common. Despite its limitations, the MMSE is widely used as a screening instrument to detect neurocognitive impairment and remains freely available for individual use.

### Recommendations

- Karnofsky performance score (KPS), neurological function, age, and individual risks and benefits should be considered for clinical decision-making. C: IV; L: A.
- Screening and prevention have no major role for patients with gliomas. C: IV; L: C.
- Patients with relevant germline variants or suspected hereditary cancer syndromes should receive genetic counselling and might subsequently be referred for molecular genetic testing. C: IV; L: C.

## Preoperative diagnostics

Brain MRI, including T2-weighted, T2-weighted fluid-attenuated inversion recovery (FLAIR) sequences and 3D T1-weighted sequences before and after application of a gadolinium-based contrast agent, is the diagnostic gold standard to detect a brain tumour^17^. Perfusion MRI and amino acid PET can help to define metabolic hotspots for specific tumour tissue sampling, a technique that can be particularly useful if biopsy rather than open resection is considered^18^. Electroencephalography can be helpful in the monitoring of tumour-associated epilepsy and in determining the cause of altered consciousness. A large number of studies has shown that cell-free tumour DNA can be detected in the plasma and cerebrospinal fluid of patients with glioma; however, the benefits of using liquid biopsies for the screening, early detection or preoperative work-up of patients with gliomas remain to be proven^19^.

### Recommendations

- The first choice of diagnostic imaging modality is MRI without and with the administration of a gadolinium-based contrast agent. C: IV; L: B.
- Pseudoprogression should be considered in patients with an increase of abnormalities on neuroimaging in the first months after local therapeutic interventions, including radiotherapy, and after experimental local treatments. C: IV; L: B.

## Preoperative management

Patient management before surgery should follow written local standard operating procedures and involve multidisciplinary discussions, ideally by a dedicated multidisciplinary tumour board including neuroradiologists and neuropathologists as well as neurosurgeons, radiation oncologists and dedicated neuro-oncologists from neurology or medical oncology services and from paediatric oncology as needed. Prior to surgery, corticosteroids can be administered to decrease symptomatic tumour-associated oedema unless primary cerebral lymphoma or inflammatory lesions are suspected. Alternative pharmacological measures, such as osmotic agents, are rarely necessary. Patients who have suffered epileptic seizures should receive anticonvulsant drugs preoperatively. Primary prophylaxis does not reduce the risk of a first seizure in patients with glioma without a history of seizures^20^.

## Tissue acquisition

Treatment decisions in patients with glioma are made based on tissue diagnosis, including the assessment of molecular markers relevant for diagnosis; therefore, upfront surgery is commonly performed with both diagnostic and therapeutic intent. The surgical management of patients with glioma should take place in high-volume specialist centres where large numbers of patients are referred to specialist neurosurgeons^21^. A decision for palliative care management without histological diagnosis should be avoided unless the risk of adverse outcomes from biopsy sampling is considered too high or if the prognosis is likely to be very unfavourable, for example, in patients with a high burden of comorbidities, large lesions with a typical radiological appearance of glioblastoma and rapid neurological deterioration. Definitive histological diagnoses aid in the counselling of patients and caregivers, even when no further tumour-specific therapy is recommended.

When microsurgical resection is not safely feasible (for example, owing to the tumour location or the impaired clinical condition of the patient), a stereotactic biopsy should be performed. Frame-based or frame-less stereotactic biopsy sampling is associated with a low risk of morbidity and a high level of diagnostic accuracy^22,23^. Serial samples of the tumour mass should be acquired along the trajectory of the biopsy needle in order to avoid sampling bias. Experienced teams can derive adequate tissue specimens for molecular profiling using these techniques^22^. IDH mutations and 1p/19q codeletion as disease-defining markers as well as *MGMT* promoter methylation^24^ are homogeneously present within tumours and, thus, the risk of sampling error for these markers is low. However, for additional markers of interest for which homogeneity has not been shown, sampling has to include different areas of the tumour; this principle applies for both stereotactic and open procedures. Intraoperative use of the fluorescent dye 5-aminolevulinic acid can be helpful to ensure adequate sampling during stereotactic biopsies^25^. Some centres prefer open biopsy approaches to ensure that sufficient tissue is obtained for any molecular studies that might be required to guide clinical decision-making.

### Recommendations

- Clinical decision-making without obtaining a tissue diagnosis should be considered only in very exceptional situations. C: IV; L: not applicable.

## Integrated histomolecular classification

**Fig. 1:**
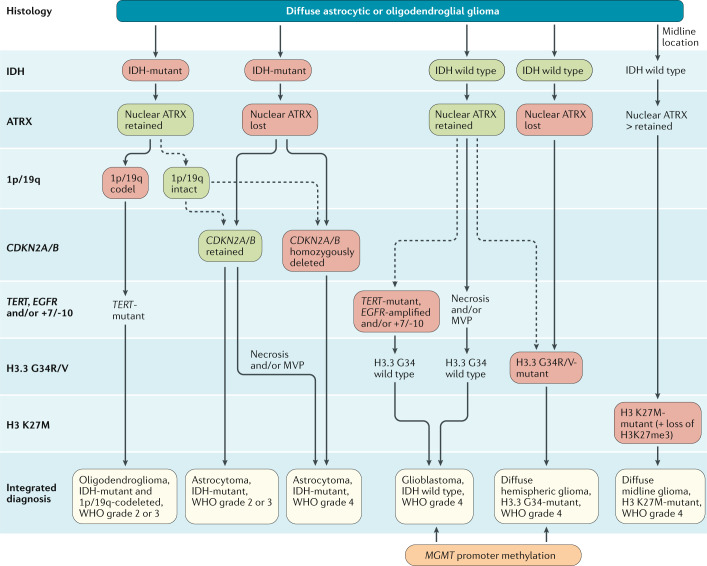
Diagnostic algorithm for the integrated classification of the major diffuse gliomas in adults. Tissue specimens obtained through biopsy sampling in patients with diffuse gliomas are routinely assessed by immunohistochemistry for the presence of R132H-mutant IDH1 and loss of nuclear ATRX. In patients aged >55 years with a histologically typical glioblastoma, without a pre-existing lower grade glioma, with a non-midline tumour location and with retained nuclear ATRX expression, immunohistochemical negativity for IDH1 R132H suffices for the classification as IDH-wild-type glioblastoma^1^. In all other instances of diffuse gliomas, a lack of IDH1 R132H immunopositivity should be followed by *IDH1* and *IDH2* DNA sequencing to detect or exclude the presence of non-canonical mutations. IDH-wild-type diffuse astrocytic gliomas without microvascular proliferation or necrosis should be tested for *EGFR* amplification, *TERT* promoter mutation and a +7/–10 cytogenetic signature as molecular characteristics of IDH-wild-type glioblastomas^2^. In addition, the presence of histone H3.3 G34R/V mutations should be assessed by immunohistochemistry or DNA sequencing to identify H3.3 G34-mutant diffuse hemispheric gliomas, in particular in young patients with IDH-wild-type gliomas (such as those <50 years of age with nuclear ATRX loss in tumour cells). Diffuse gliomas of the thalamus, brainstem or spinal cord should be evaluated for histone H3 K27M mutations and loss of nuclear K27-trimethylated histone H3 (H3K27me3) to identify H3 K27M-mutant diffuse midline gliomas. The presence and absence of the diagnostically most relevant molecular alterations for each tumour type are highlighted with red and green boxes. MVP, microvascular proliferation.

Intraoperative assessment of cytological specimens or frozen sections ensures that sufficient tumour tissue is obtained to establish a diagnosis. Tumour tissue is formalin fixed and embedded in paraffin for histological and immunohistochemical staining as well as for molecular genetic and cytogenetic studies. If possible, some tumour tissue should be cryopreserved for molecular assessments that require high-quality DNA and RNA samples. The diagnostic process should follow the WHO classification of 2016 (ref.^1^) and the subsequent recommendations from cIMPACT-NOW^2–4^. Accordingly, glioma classification integrates histological tumour typing and grading as well as analyses of molecular markers (Fig. 1). The term ‘not otherwise specified’ was introduced to refer to gliomas that were not tested for markers relevant to the diagnosis of specific subtypes or for which testing was inconclusive^1^.

On the basis of the 2016 WHO classification and cIMPACT-NOW recommendations, the following molecular biomarkers are central to categorizing diffuse gliomas in adults: IDH mutation, 1p/19q co-deletion, histone H3 K27M mutation, histone H3.3 G34R/V mutation, *TERT* promoter mutation, *EGFR* gene amplification, chromosome 7 gain combined with chromosome 10 loss (the +7/–10 signature), and homozygous deletions on 9p21 involving the *CDKN2A* and *CDKN2B* gene loci (*CDKN2A/B* homozygous deletion) (Table 1). Missense mutations in codon 132 of *IDH1* or codon 172 of *IDH2* are the defining molecular feature of IDH-mutant astrocytomas and are associated with the glioma CpG island methylator phenotype (G-CIMP). Diffuse gliomas corresponding histologically to WHO grade 2 or 3 that are immunohistochemically negative for IDH1 R132H should be sequenced for less common *IDH1* and for *IDH2* mutations. IDH-mutant astrocytomas usually also have loss of nuclear expression of ATRX and mutations in *TP53* but, by definition, lack 1p/19q codeletion^1^. Indeed, the detection of nuclear ATRX loss in an IDH-mutant glioma is sufficient for the diagnosis of an astrocytic lineage tumour without the need for 1p/19q codeletion analysis. By contrast, retained nuclear ATRX positivity in an IDH-mutant glioma should prompt analysis for 1p/19q codeletion in order to distinguish IDH-mutant astrocytoma from IDH-mutant and 1p/19q-codeleted oligodendroglioma. ATRX immunohistochemistry is not necessary if IDH mutation and 1p/19q codeletion status are captured within one more extensive molecular marker panel assay. IDH-mutant astrocytomas are now stratified into three WHO grades: astrocytoma, IDH-mutant, WHO grade 2; astrocytoma, IDH-mutant, WHO grade 3 (instead of anaplastic astrocytoma, IDH-mutant, WHO grade 3); and astrocytoma, IDH-mutant, WHO grade 4 (replacing the former term ‘glioblastoma, IDH-mutant, WHO grade 4’)^3^. The term ‘glioblastoma’ is no longer used to refer to IDH-mutant astrocytic gliomas because these tumours are biologically distinct from the much more common IDH-wild-type glioblastomas, although their histological appearance is similar^3^. In addition to the established histological features, such as the presence of necrosis and/or microvascular proliferation, homozygous *CDKN2A/B* deletion is indicative of a poor prognosis^26^ and is a marker of WHO grade 4 IDH-mutant astrocytomas^3^. As the diagnostic term ‘astrocytoma, IDH-mutant’ can be associated with different tumour grades and the roman numerals II and III are easily confused, cIMPACT-NOW recommended the use of Arabic numerals for the WHO-based grading of these tumours^3^. In line with the sixth update of the cIMPACT-NOW recommendations^4^, in these evidence-based guidelines we use Arabic numerals for WHO grades.

**Table 1:** Molecular markers for the diagnosis and management of gliomas

Molecular marker	Biological function of affected genes	Diagnostic roles
IDH1 R132 or IDH2 R172 mutation	Gain-of-function mutation	Distinguishes diffuse gliomas with IDH mutation from IDH-wild-type glioblastomas and other IDH-wild-type gliomas
1p/19q codeletion	Inactivation of putative tumour suppressor genes on 1p (such as *FUBP1*) and 19q (such as *CIC*)	Distinguishes oligodendroglioma, IDH-mutant and 1p/19q-codeleted from astrocytoma, IDH-mutant
Loss of nuclear ATRX	Cell proliferation and promotion of cellular longevity by alternative lengthening of telomeres	Loss of nuclear ATRX in an IDH-mutant glioma is diagnostic for astrocytic lineage tumours
Histone H3 K27M mutation	Histone H3.3 (*H3F3A*) or histone H3.1 (*HIST1H3B*/*C*) missense mutation affecting epigenetic regulation of gene expression	Defining molecular feature of diffuse midline glioma, H3 K27M-mutant
Histone H3.3 G34R/V mutation	Histone mutation affecting epigenetic regulation of gene expression	Defining molecular feature of diffuse hemispheric glioma, H3.3 G34-mutant
*MGMT* promoter methylation	DNA repair	None, but is a predictive biomarker of benefit from alkylating chemotherapy in patients with IDH-wild-type glioblastoma
Homozygous deletion of *CDKN2A/CDKN2B*	Encode cyclin-dependent kinase inhibitors 2A and 2B and tumour suppressor ARF, which function as regulators of Rb1 and p53-dependent signalling	A marker of poor outcome and WHO grade 4 disease in IDH-mutant astrocytomas
*EGFR* amplification	Cell proliferation, invasion and resistance to induction of apoptosis	*EGFR* amplification occurs in ~40–50% of glioblastoma, IDH wild type Molecular marker of glioblastoma,IDH wild type, WHO grade 4 (ref.^3^)
*TERT* promotor mutation	Cell proliferation; promotes cellular longevity by increasing TERT expression	*TERT* promoter mutation occurs in ~70% of glioblastoma, IDH wild type and >95% of oligodendroglioma, IDH-mutant and 1p/19q-codeleted Molecular marker of glioblastoma, IDH wild type, WHO grade 4 (ref.^3^)
+7/–10 cytogenetic signature	Gain of chromosome 7 (harbouring genes encoding, among others, PDGFA and EGFR) combined with loss of chromosome 10 (harbouring genes including *PTEN* and *MGMT*)	Molecular marker of glioblastoma, IDH wild type, WHO grade 4 (ref.^3^)
*BRAF*^V600E^ mutation	Oncogenic driver mutation leading to MAPK pathway activation	Rare in adult diffuse gliomas but amenable to pharmacological intervention

Special attention should be given to diffuse astrocytomas in the brainstem or cerebellum with histologies corresponding to WHO grades 2, 3 and 4. Among infratentorial astrocytomas, the frequency of non-canonical IDH mutations is ~80%, in contrast with <10% in those of the supratentorial compartment^27,28^. Infratentorial diffuse gliomas therefore tend to be classified incorrectly if examined by IDH1 R132H immunohistochemistry only; accordingly, DNA sequencing for rare mutations in *IDH1* and *IDH2* is required. In addition, infratentorial IDH-mutant astrocytomas have a loss of nuclear ATRX expression as well as *MGMT* promoter methylation in only ~50% of patients^27,28^.

Oligodendroglial tumours are defined as IDH-mutant gliomas that also harbour 1p/19q codeletion^1^ and are stratified into WHO grade 2 or 3 tumours based on the absence or presence of histological features of anaplasia. The role of molecular alterations in the grading of these tumours has not been defined. However, similar to IDH-mutant diffuse astrocytomas, the homozygous deletion of *CDKN2A* at 9p21 has been associated with shorter survival durations^29^. Oligoastrocytomas lack characteristic genetic profiles and are no longer considered as a distinct glioma subtype.

Astrocytic gliomas with a wild-type IDH and histone H3 status and with necrosis and/or microvascular proliferation are classified as IDH-wild-type, WHO grade 4 glioblastomas^1^. In the absence of necrosis or microvascular proliferation, such tumours should be evaluated for glioblastoma-associated genetic alterations, in particular *EGFR* gene amplifications, *TERT* promoter mutations and/or the +7/–10 signature^2^. If one or more of these alterations is detected, these tumours are classified as IDH-wild-type glioblastomas given their association with a poor prognosis, even in the absence of necrosis and microvascular proliferation^1,30^. IDH-wild-type diffuse astrocytomas without any of these alterations, which cannot be assigned to other entities (for example, on the basis of DNA methylation profiling) are more often seen in paediatric, adolescent or young adult patients and constitute rare glioma variants that require further molecular assessment^31^.

H3 K27M-mutant, WHO grade 4 diffuse midline gliomas are defined as a diffuse glioma located in midline structures, such as the thalamus, pons, brainstem and spinal cord, and carrying a lysine-to-methionine mutation at amino acid 27 of histone H3.3 (encoded by *H3F3A*) or histone H3.1 (encoded by *HIST1H3B* and *HIST1H3C*)^1^. H3 K27M-mutant diffuse midline gliomas are typically positive for nuclear immunostaining of H3 K27M with the corresponding loss of nuclear staining for K27-trimethylated histone H3 (H3K27me3), which together serve as immunohistochemical markers of this tumour type. H3.3 G34-mutant, WHO grade 4 diffuse hemispheric glioma has been proposed as a new subtype of malignant glioma, characterized by missense mutations affecting codon 34 of *H3F3A*^4^^,^^31^.

*MGMT* promoter methylation has limited diagnostic value but can guide treatment decisions on the use of chemotherapy with alkylating agents for patients with glioblastoma or other IDH-wild-type gliomas^32^. As outlined below, *MGMT* promoter methylation enables the prediction of benefit from alkylating agents in patients with these tumours. *MGMT* promoter methylation status should be tested using methylation-specific PCR, pyrosequencing or methylation arrays (such as the MGMT-STP27 model)^33^. However, challenges remain, including: (1) establishing reliable *MGMT* promoter methylation status assays that can be used with high interlaboratory agreement, and (2) estimating the effect of limited *MGMT* promoter methylation, an intermediate state between the non-methylated and methylated phenotypes, on outcomes^33^. Immunocytochemistry is not an adequate method to determine the *MGMT* promoter methylation status^34^.

Next-generation sequencing-based gene panels could enable the assessment of all or most genetic and chromosomal aberrations relevant for diagnosis using a single assay^35,36^. In addition, array-based DNA methylation profiling has emerged as a powerful novel diagnostic method that is independent of histology and useful in the routine diagnostic work-up^37^. Moreover, RNA sequencing-based approaches present a promising approach for the detection of oncogenic gene fusions with diagnostic and/or predictive value that can be found in rare subsets of diffuse gliomas, mainly IDH-wild-type glioblastomas^38,39^. Overall, molecular diagnostic algorithms for patients with glioma (Fig. 1) should be standardized and should not result in delays in the administration of radiotherapy or tumour-specific pharmacotherapy.

### Recommendations

- Glioma classification should follow the most recent WHO Classification of Tumors of the Central Nervous System^1^, complemented by cIMPACT-NOW updates^2–4^. C: IV; L: B.
- Immunohistochemistry for mutant IDH1 R132H protein and nuclear expression of ATRX should be performed routinely in the diagnostic assessment of diffuse gliomas. C: IV; L: B.
- If immunohistochemistry for IDH1 R132H is negative, sequencing of *IDH1* codon 132 and *IDH*2 codon 172 should be conducted in all WHO grade 2 and 3 diffuse astrocytic and oligodendroglial gliomas as well as in all glioblastomas of patients aged <55 years to enable integrated diagnoses according to the WHO classification and to guide treatment decisions. C: IV; L: B.
- 1p/19q codeletion status should be determined in all IDH-mutant gliomas with retained nuclear expression of ATRX. C: II; L: B.
- *MGMT* promoter methylation status should be determined in glioblastoma, notably in elderly or frail patients, to aid in decision-making for the use of temozolomide. C: I; L: B.
- *CDKN2A/B* homozygous deletions should be explored in IDH-mutant astrocytomas. C: IV; L: B.
- Combined chromosome 7 gain and chromosome 10 loss (+7/–10 signature), *EGFR* amplification and *TERT* promoter mutation should be tested in IDH-wild-type diffuse gliomas lacking microvascular proliferation and necrosis as histological features of WHO grade 4 to allow for a diagnosis of IDH-wild-type glioblastoma. C: IV; L: B.
- Assessment of H3 K27M status should be done in diffuse gliomas involving the midline. C: IV; L: B.
- *BRAF*^V600^ mutations might be assessed in IDH-wild-type diffuse gliomas. C: IV; L: C.

## Therapy — general recommendations

### Prognostic factors

Younger age and better performance status at diagnosis are major therapy-independent prognostic factors associated with favourable outcomes in adults with glioma^7^. Furthermore, molecular genetic factors, notably 1p/19q codeletion and IDH mutation status, had a strong prognostic value in the classification of gliomas in the past but, since 2016, have become disease-defining features and are therefore no longer prognostic within a given disease subtype. As a result, *MGMT* promoter methylation status has become the single most important prognostic factor in an era in which the vast majority of adults with glioma are treated with alkylating agent-based chemotherapy.

### Surgical therapy

The therapeutic goal of surgery is to remove as much tumour tissue as safely feasible using microsurgical techniques, without compromising neurological function. Several tools, including surgical navigation systems housing functional MRI or diffusion tensor imaging datasets and intraoperative MRI, ultrasonography, functional monitoring and fluorescence-based visualization of tumour tissue with 5-aminolevulinic acid, help in reducing postoperative residual tumour volumes while keeping the risk of new neurological deficits low^40^. The use of evoked potentials, electromyography or brain mapping in awake patients under local anaesthesia to monitor and preserve language and cognition facilitates resections in eloquent areas^41^. Preventing new permanent neurological deficits that might jeopardize independence, reduce quality of life (QOL) and increase the risk of additional complications that might, in turn, delay or preclude further therapy is more important than the extent of resection because diffuse gliomas are not cured by surgery. Neurological deficits that occur because of surgery can sometimes be predicted preoperatively. In exceptional situations, anticipated minor deficits (such as quadrantanopia) might be deemed acceptable but only after a thorough process of shared decision-making^42^. Patients and their caregivers should also be informed that neurosurgery is always associated with some unpredictable risks. Postoperative deficits owing to emerging surgical complications are a negative prognostic factor that can interfere with further treatment and health-related QOL is of high priority to patients and their caregivers^43^. The extent of resection should be assessed within 24–48 hours of surgery through MRI (or CT if MRI is not possible), without and with contrast; MRI should include diffusion-weighted sequences to enable the detection of perioperative ischaemia^44^.

The role of the extent of resection and residual tumour volume as prognostic factors remains controversial within the neuro-oncology community because randomized controlled trials (RCTs) addressing this question are very difficult to perform, and almost no such trials exist. A lesser extent of resection and larger post-surgical residual tumour volumes are negative prognostic factors across gliomas of all grades and subtypes^45,46^. These observations have resulted in the multitude of technical developments to maximize the extent of resection summarized above. Nevertheless, whether and why the extent of resection truly matters remain controversial questions. First, rather than the percentage of extent of resection, clinicians might need to consider the absolute volume of remaining tumour tissue, including both enhancing and non-enhancing tumour tissue^45–47^. Second, early (<3 weeks) as opposed to later (3–5 weeks) initiation of postsurgical radiotherapy does not correlate with improved overall survival (OS)^48^. This finding is unexpected because one might predict that a longer time interval between surgery and start of radiotherapy would favour regrowth of the tumour and thus confer a survival disadvantage^47^. Third, evidence indicates that resectable tumours have a different biology that is overall less malignant than that of non-resectable tumours, which challenges the causal relationship between extent of surgery and survival. For example, in a prospective evaluation of the effect of surgical resection on survival after controlling for IDH status, the rate of gross total resection was higher in patients with IDH-mutant tumours than in those with IDH-wild-type tumours^49^. Indeed, retrospective data indicate that biopsy is more often the type of first surgery in patients with IDH-wild-type tumours than in patients with IDH-mutant tumours^47^. Attributing the longer survival durations associated with IDH-mutant versus IDH-wild-type tumours to the rate of gross total resection would therefore probably not be the correct conclusion. With these considerations, we do not intend to discourage efforts to achieve gross total resection but rather to acknowledge the limitations of data from retrospective uncontrolled studies.

### Recommendations

- The extent of resection is a prognostic factor and thus, efforts at obtaining complete resections are justified across all glioma entities. C: IV; L: B.
- In the current surgical approach to gliomas, the prevention of new permanent neurological deficits has higher priority than the extent of resection. C: IV; L: C.

### Radiotherapy

The goal of radiotherapy is to improve local control without inducing neurotoxicity. Indeed, radiotherapy delayed neurological deterioration and increased survival in several early clinical trials conducted in the past century^50,51^. The timing, dosing and scheduling of radiotherapy are determined by the disease subtype and prognostic factors, including age, KPS and residual tumour volume. Radiotherapy should start within 3–5 weeks after surgery^48^ and is commonly administered at 50–60 Gy in 1.8–2 Gy daily fractions. No evidence suggests additional benefit from high-dose versus low-dose radiation in patients with WHO grade 2 gliomas^52^ and, for those with higher WHO grade tumours, no data from randomized studies support the use of doses >60 Gy (ref.^53^). Hypofractionated radiotherapy with a higher dose per fraction and a lower total dose (for example, 15 × 2.67 Gy) is appropriate in older patients (>65–70 years of age) and in those with a poor prognosis (typically defined by a KPS of <70)^54^.

The area of the surgical bed plus the residual tumour area identified on T1-weighted, T2-weighted and FLAIR MRI sequences is defined as the gross tumour volume. To account for microscopic invasion, a margin of 1.0–2.0 cm is added to create the clinical target volume, which is generally modified to include abnormalities visualized on the basis of T2-weighted or FLAIR signals (for example, oedema) and constrained to anatomical barriers such as ventricles, tentorium and falx. Finally, another margin, usually of 0.3–0.5 cm, is added to enable for uncertainties in patient set-up and treatment delivery, generating the planning target volume^55^. The use of amino acid PET using tracers such as [^11^C-methyl]-l-methionine or O-(2-[^18^F]-fluoroethyl)-l-tyrosine to improve target delineation for radiotherapy has been evaluated in clinical trials but is not currently part of standard practice^18^. Structures at higher risk of toxicity from radiotherapy, including the optic nerves, optic chiasm, retinae, lenses, brainstem, pituitary, cochleae and hippocampi, should be delineated. Modern, highly conformal radiation techniques, including intensity-modulated radiotherapy for newly diagnosed tumours and stereotactic radiotherapy and radiosurgery for recurrent tumours, could provide superior target coverage and sparing of non-malignant brain tissue. Proton or heavy ion radiotherapy might be options to consider for patients with tumours close to brain regions at risk or in those with a favourable prognosis in order to avoid delayed toxicities, but RCTs are required to determine the tolerability, safety and efficacy of these approaches compared with standard radiotherapy^56,57^. Accurate patient positioning is required for all highly conformal approaches and is achieved with reproducible immobilization and digital imaging during treatment. Interstitial brachytherapy approaches have been investigated over many years as an alternative to external beam treatment but have not yet been shown to have an application in routine practice^58^. An MRI scan scheduled 3–4 weeks after completion of radiotherapy provides a new baseline to monitor the further course of disease.

### Pharmacotherapy

Haematology, hepatic and renal laboratory values within the normal physiological ranges and exclusion of major lung or heart disease or infection are required prior to and during most pharmacological treatments for patients with glioma. Most patients with glioma receive chemotherapy with alkylating agents at some point in their disease course. Temozolomide, an oral DNA alkylating agent that penetrates the blood–brain barrier, is the most commonly used drug in glioma treatment. This agent has a favourable safety profile, with myelosuppression, notably thrombocytopenia, as its main dose-limiting toxicity^59^. Hepatic function also needs to be monitored regularly in patients receiving temozolomide. In contrast to temozolomide, alkylating agents from the nitrosourea class, such as lomustine, carmustine, nimustine or fotemustine, cause delayed (4–6 weeks) rather than early (2–3 weeks) and more often cumulative leukopenia and thrombocytopenia. Notably, the latter can necessitate treatment interruptions, dose reductions or even discontinuation and consideration of alternative treatments. Pulmonary fibrosis has been observed mainly with carmustine and is rare with lomustine^60^. Lomustine is often combined with procarbazine and vincristine in a regimen referred to as PCV. Carmustine wafers implanted into the post-surgical cavity provided a modest OS benefit in patients with newly diagnosed WHO grade 3 or 4 gliomas or recurrent glioblastoma^61,62^; however, in the pivotal trial of this approach, patient outcomes were not statistically significantly different after patients with WHO grade 3 tumours (the majority of which are now known to be IDH-mutant) were excluded from the survival analysis. The benefit from alkylating agent chemotherapy demonstrated in various RCTs (described later) has to be weighed against the potential long-term toxicities and the risk of inducing a hypermutator phenotype that is associated with a more malignant phenotype, in particular in patients with IDH-mutant gliomas, who have a longer life expectancy^63,64^.

Bevacizumab, an anti-VEGF antibody, is approved for the treatment of recurrent glioblastoma in the USA, Canada, Switzerland and several other countries outside the European Union, but no OS benefit has been demonstrated from its use^65–67^. Patients with glioma receiving systemic therapy should carry a documentation of treatment, including laboratory results and information on complications and contraindications, to facilitate follow-up and to provide information to physicians in an emergency setting. Clinical centres managing patients with glioma should generate standard operating procedures and instructions for standardized application of chemotherapy as well as for the management of adverse events and complications from treatment.

### Monitoring and follow-up assessments

Watch-and-wait strategies without histological verification carry the risk of underestimating the grade of malignancy when determined using only neuroimaging and thus require initial intervals of only 2–3 months between scans. In addition to clinical examination, MRI is the standard diagnostic measure for the evaluation of disease status or treatment response, using Response Assessment in Neuro-Oncology (RANO) criteria^68–70^ and identical MRI protocols according to published recommendations^71^. After the completion of therapy, an initial interval between scans of 2–6 months is common practice for most patients depending on the disease histology but longer intervals might be appropriate in cases of durable disease control and more benign tumours. Careful consideration of not only the most recent MRI scan but also of the complete disease trajectory is required, specifically in patients with slow-growing untreated lesions^72^. Conversely, in the event of suspected disease progression, short-term control MRI within 4–8 weeks might be reasonable to confirm progression. Pseudoprogression (typically after chemoradiotherapy or immunotherapy) and pseudoresponse (for example, after anti-angiogenic therapy) are most likely to occur during the first 3 months of treatment but can also occur later^70^. Particular attention is needed when interpreting scans during this period; in case of doubt, rescanning after shorter intervals (4–8 weeks) is a pragmatic approach. Perfusion MRI and amino acid PET might help to distinguish pseudoprogression from true disease progression^73^. Biopsy sampling is not always informative because viable tumour cells are regularly detected but their presence does not rule out pseudoprogression.

As for other non-curable diseases, patients with gliomas should be offered counselling by specialized psychologists or nurses and palliative care specialists. The need for occupational, speech and physical therapy as well as for counselling for social support should be assessed^74^.

## Therapy — specific recommendations

### IDH-mutant and 1p/19q-codeleted oligodendroglioma, WHO grade 2

**Fig. 2:**
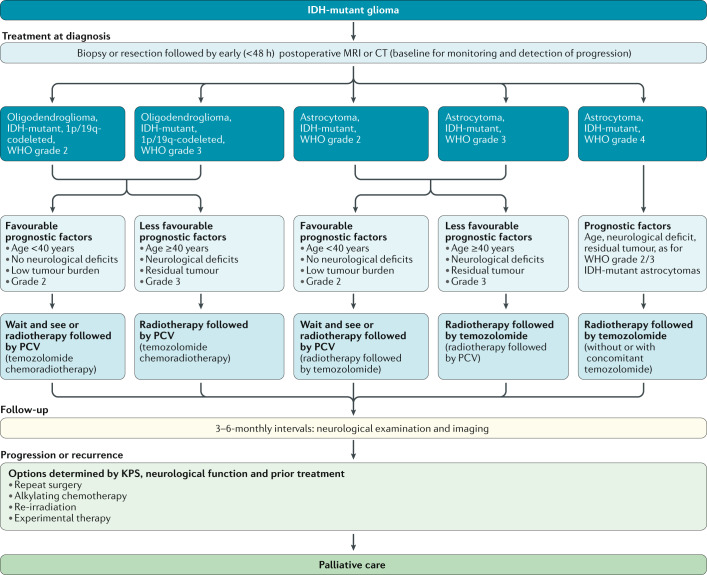
Clinical pathway for IDH-mutant gliomas. KPS, Karnofsky performance status; PCV, procarbazine, lomustine and vincristine.

Surgery is the primary treatment modality for patients with gliomas of this subtype. Following surgery, watch-and-wait strategies are justified in those with gross total resection and potentially also in younger patients (<40 years of age) with incomplete resection if the tumour has not yet caused neurological deficits beyond symptomatic epilepsy. If further treatment beyond surgery is deemed necessary, the standard of care is radiotherapy followed by PCV^75^. The use of chemotherapy alone remains investigational but might be an option to reduce the risk of late cognitive deficits in patients with large tumours owing to the favourable outcomes of this patient population relative to those with other subtypes^76,77^. The choice of treatment at recurrence depends on the initial treatment (Table 2, Fig. 2).

**Table 2:** Key treatment recommendations for adult patients with common diffuse gliomas

Tumour type^a^	Treatment at diagnosis^b^	Treatment at progression or recurrence^c,d^	Comments
Astrocytoma, IDH-mutant, WHO grade 2, including gemistocytic astrocytoma, IDH-mutant, WHO grade 2 (cIMPACT-NOW, previously diffuse astrocytoma, IDH-mutant, WHO grade 2)	Wait-and-see or radiotherapy (50–54 Gy in 1.8–2 Gy fractions) followed by PCV (or temozolomide chemoradiotherapy)	Temozolomide (or nitrosourea)	RTOG 9802 (ref.^75^) and per extrapolation from WHO grade 3 tumours^88^
Diffuse astrocytoma, IDH wild type, WHO grade 2^a,e^	Wait-and-see (?); radiotherapy (50–54 Gy in 1.8–2 Gy fractions); radiotherapy followed by PCV or temozolomide chemoradiotherapy (by *MGMT* status?)	Temozolomide; nitrosourea; bevacizumab^f^	Heterogeneous group of tumours awaiting further subclassification^e^
Diffuse astrocytoma, NOS^g^, WHO grade 2	See astrocytoma, IDH-mutant, WHO grade 2	See astrocytoma, IDH-mutant, WHO grade 2	Per extrapolation because most of these tumours carry IDH mutations
Astrocytoma, IDH-mutant, WHO grade 3 (cIMPACT-NOW, previously anaplastic astrocytoma, IDH-mutant, WHO grade 3)	Radiotherapy (54–60 Gy in 1.8–2 Gy fractions) followed by temozolomide (or wait-and-see)	Nitrosourea; temozolomide rechallenge	^88^
Anaplastic astrocytoma, IDH wild type, WHO grade 3	Radiotherapy (54–60 Gy in 1.8–2 Gy fractions); temozolomide chemoradiotherapy, by *MGMT* promoter methylation status (?)	Temozolomide rechallenge; nitrosourea; bevacizumab^f^	Per extrapolation from IDH-wild-type glioblastoma^32,59^
Anaplastic astrocytoma, NOS, WHO grade 3	See astrocytoma, IDH-mutant, WHO grade 3	Nitrosourea; temozolomide rechallenge	Per extrapolation because most of these tumours carry IDH mutations
Oligodendroglioma, IDH-mutant and 1p/19q-codeleted, WHO grade 2	Wait-and-see; radiotherapy (50–54 Gy in 1.8–2 Gy fractions) followed by PCV	Temozolomide	Per extrapolation from WHO grade 3 tumours^79,80^ and RTOG 9802 (ref.^75^)
Oligodendroglioma, NOS, WHO grade 2	See oligodendroglioma, IDH-mutant and 1p/19q-codeleted, WHO grade 2	See oligodendroglioma, IDH-mutant and 1p/19q-codeleted, WHO grade 2	Per extrapolation because most of these tumours carry IDH mutations
Oligodendroglioma, IDH-mutant and 1p/19q-codeleted, WHO grade 3 (cIMPACT-NOW, previously anaplastic oligodendroglioma, IDH-mutant and 1p/19q-codeleted, WHO grade 3)	Radiotherapy (54–60 Gy in 1.8–2 Gy fractions) followed by PCV (or wait-and-see)	Temozolomide	^79,80^
Anaplastic oligodendroglioma, NOS, WHO grade 3	See oligodendroglioma, IDH-mutant and 1p/19q-codeleted, WHO grade 3	See oligodendroglioma, IDH-mutant and 1p/19q-codeleted, WHO grade 3	Per extrapolation because most of these tumours carry IDH mutations
Oligoastrocytoma, NOS, WHO grade 2	Wait-and-see; radiotherapy (50–54 Gy in 1.8–2 Gy fractions) followed by PCV	Temozolomide	Per extrapolation from WHO grade 3 tumours^79,80^ and RTOG 9802 (ref.^75^)
Anaplastic oligoastrocytoma, NOS, WHO grade 3	Radiotherapy (54–60 Gy in 1.8–2 Gy fractions) followed by PCV (or wait-and-see)	Temozolomide	^79,80^
Astrocytoma, IDH-mutant, WHO grade 4 (cIMPACT-NOW, previously glioblastoma, IDH-mutant, WHO grade 4)	Temozolomide chemoradiotherapy (54–60 Gy in 1.8–2 Gy fractions) (potentially without concomitant temozolomide)	Nitrosourea; temozolomide rechallenge; bevacizumab^f^	Per extrapolation from IDH-mutant anaplastic astrocytoma^88^ or from glioblastoma^59^
Glioblastoma, IDH wild type, WHO grade 4; giant cell glioblastoma; gliosarcoma; epithelioid glioblastoma	Temozolomide chemoradiotherapy (54–60 Gy in 1.8–2 Gy fractions); for patients aged >65–70 years and MGMT unmethylated tumours, radiotherapy (40 Gy in 2.67 Gy fractions); for patients aged >65–70 years and MGMT methylated tumours, temozolomide chemoradiotherapy or temozolomide^h^	Nitrosourea; temozolomide rechallenge; bevacizumab^f^; radiotherapy (for patients not previously treated with radiotherapy)	^59,94,96^^–^^98^
Glioblastoma, NOS, WHO grade 4	Temozolomide chemoradiotherapy (54–60 Gy in 1.8–2 Gy fractions); for patients aged >65–70 years and MGMT unmethylated tumours, radiotherapy (40 Gy in 2.67 Gy fractions); for patients aged >65–70 years and MGMT methylated tumours, temozolomide chemoradiotherapy or temozolomide	Nitrosourea; temozolomide; rechallenge; bevacizumab^c^; radiotherapy (for patients not previously treated with radiotherapy)	^59^
Diffuse midline glioma, H3 K27M-mutant, WHO grade 4	Radiotherapy (54–60 Gy in 1.8–2 Gy fractions); temozolomide chemoradiotherapy	Nitrosourea; temozolomide rechallenge; bevacizumab^f^	Per extrapolation^59^
Diffuse hemispheric glioma, H3.3 G34-mutant, WHO grade 4	Temozolomide chemoradiotherapy	Nitrosourea; temozolomide rechallenge; bevacizumab^c^	Per extrapolation^59^

According to the 2016 WHO classification^1^ and cIMPACT-NOW updates 3, 5 and 6 (refs^2–4^). NOS, not otherwise specified; PCV, procarbazine, lomustine and vincristine. ^a^Provisional and NOS tumour categories are indicated in italics. ^b^Maximum safe resection is recommended whenever feasible in all patients with newly diagnosed gliomas. ^c^Second surgery should always be considered but clinical benefit might be limited to patients in whom a gross total resection can be achieved. Indications for reirradiation remain controversial. ^d^Re-exposure to temozolomide and nitrosoureas is associated with limited activity in tumours without *MGMT* promoter methylation. ^e^Diffuse astrocytomas, IDH wild type are a heterogeneous tumour group that should be further molecularly characterized to separate malignant tumours with molecular features of IDH-wild-type glioblastoma from indolent tumours (for example, corresponding to paediatric-type diffuse gliomas). ^f^Depending on local availability. ^g^Management recommendations for NOS categories are included, but evidence is low. Of note, most practice-defining trials included herein enrolled patients prior to the 2016 revision of the WHO classification. ^h^Tumour-treating fields remain controversial when applied in the temozolomide maintenance setting despite a phase III trial with positive results^101^ and are not widely available in Europe.

### IDH-mutant and 1p/19q-codeleted oligodendroglioma, WHO grade 3

In this subtype, the extent of resection is a prognostic factor^78^. The distinction of two grades (2 and 3) of IDH-mutant, 1p/19q-codeleted gliomas remains controversial and, accordingly, watch-and-wait strategies after complete resection can also be considered for younger patients (<40 years of age) with WHO grade 3 tumours, specifically for those without homozygous *CDKN2A/B* deletion, although only after gross total resection and in the absence of neurological deficits. Two large RCTs showed that the addition of PCV, either prior to or after radiotherapy, in the first-line of treatment approximately doubled the OS^79,80^. Although these results stem from analyses of small cohorts of patients, both studies showed similar results, thus validating the findings and defining the current standard of care. Important open questions include: (1) whether neurocognitive function and health-related QOL are preserved in long-term survivors treated with radiotherapy and PCV^81^, and (2) whether the same improvement in OS could be achieved with temozolomide-based chemoradiotherapy. Long-term results from the NOA-04 trial showed that chemotherapy alone (either PCV or temozolomide) is not superior to radiotherapy alone in any molecular subgroup of anaplastic glioma, thus indicating that alkylating agent-based chemotherapy alone is unlikely to result in the same outcome as radiotherapy followed by PCV^82^. The modified CODEL trial^83^ will address whether temozolomide-based chemoradiotherapy is similarly effective as radiotherapy followed by PCV.

The choice of treatment at progression is influenced by the choice of and response to first-line therapy (Fig. 2). Second surgery should always be considered. If neither radiotherapy nor alkylating agents are options owing to ineffectiveness or intolerance in the first-line setting, bevacizumab can be used for symptom control; however, the antitumour efficacy of bevacizumab is unknown and no evidence supports its combination with cytotoxic agents in this setting.

### IDH-mutant astrocytoma, WHO grade 2

Most WHO grade 2 astrocytomas harbour IDH mutations. Gemistocytic astrocytoma is a distinct variant of IDH-mutant astrocytoma, WHO grade 2. Maximal surgical resection, if safely feasible, is the best initial therapeutic approach^84^. Watch-and-wait strategies without the establishment of an integrated diagnosis should only be considered in exceptional situations, even for patients with incidentally discovered lesions. Younger patients (pragmatic cut-off ~40–45 years of age) who are asymptomatic or with seizures only, can be managed through observation alone after gross total resection. Involved-field radiotherapy (50 Gy in 1.8 Gy fractions) should be considered for patients with incomplete resection and/or for patients aged >40 years. Early radiotherapy (as opposed to radiotherapy after disease progression) has been shown to prolong progression-free survival (PFS) but not OS^85^. The use of chemotherapy alone as frontline therapy remains investigational but might be an option if radiotherapy is not feasible, for example, in patients with large tumours. However, the PFS is probably shorter with temozolomide than with radiotherapy in patients with IDH-mutant, grade 2 diffuse astrocytomas^86^. The RTOG 9802 trial reported a major prolongation of OS with the addition of PCV polychemotherapy to radiotherapy (54 Gy), from 7.8 years to 13.3 years in patients with high-risk WHO grade 2 gliomas who were 18–39 years of age and had undergone a subtotal resection or biopsy or in those aged ≥40 years^75^. This benefit was reported across histological subgroups and, although cohort sizes were small, benefit was observed in patients with either IDH-mutant astrocytomas or oligodendrogliomas but not in those with IDH-wild-type tumours^87^. Thus, radiotherapy followed by PCV constitutes the standard of care for patients with WHO grade 2 IDH-mutant astrocytomas deemed to require post-surgical treatment.

Treatment at progression depends on neurological status, patterns of progression and first-line therapy (Fig. 2). Second surgery should always be considered, usually followed by radiotherapy in patients who had not previously received irradiation, or by alkylating agent-based chemotherapy. Temozolomide is often preferred over PCV in this setting owing to its favourable safety profile and ease of administration.

### IDH-mutant astrocytoma, WHO grade 3

The standard of care for patients with this disease subtype is maximal surgical resection or biopsy followed by radiotherapy at 60 Gy in 1.8–2 Gy fractions (Table 1). This approach was established largely based on trials in which subgroups of patients with WHO grade 3 tumours were pooled with those with glioblastomas. The NOA-04 trial showed similar PFS and OS with PCV or temozolomide alone versus radiotherapy alone^78,82^. The EORTC 26053 trial (CATNON) of radiotherapy alone, with concomitant or maintenance temozolomide or with both concomitant and maintenance temozolomide showed a significant prolongation of OS in patients receiving radiotherapy followed by 12 cycles of maintenance temozolomide and, thus, this approach should be considered standard of care; however, the role of concomitant temozolomide remains uncertain^88^. Indeed, updated data from CATNON indicate that concomitant temozolomide provides limited improvement to the overall favourable outcomes associated with maintenance chemotherapy and, more importantly, that only patients with IDH-mutant tumours derive benefit from chemotherapy (either as maintenance or concomitantly)^89^.

First-line therapy informs the choice of treatment in the recurrent disease setting (Fig. 2). Second surgery should be considered for all patients. For those with disease relapse after radiotherapy, re-irradiation after a minimum interval of ~12 months following the first course of radiotherapy is an option, although tumour size and patterns of recurrence limit the option of re-irradiation and the overall efficacy of this strategy remains uncertain in the absence of data from RCTs. Alkylating agent-based chemotherapy should be considered for patients who have not received previous chemotherapy and with disease progression after radiotherapy. Temozolomide and nitrosoureas are probably equally effective in this setting^90,91^. Adding bevacizumab to temozolomide prolongs neither PFS nor OS durations in patients with contrast-enhancing recurrent IDH-mutant gliomas without 1p/19q codeletion^14^.

### Recommendations

- The standard of care for IDH-mutant astrocytomas, WHO grade 2 requiring further treatment includes resection as feasible or biopsy followed by involved field radiotherapy and maintenance PCV polychemotherapy (RTOG 9802)^75^. C: II; L: B.
- The standard of care for IDH-mutant astrocytomas, WHO grade 3 includes resection as feasible or biopsy followed by involved field radiotherapy and maintenance temozolomide (CATNON)^88^. C: II; L: B.
- Patients with IDH-mutant and 1p/19q-codeleted oligodendrogliomas, WHO grade 2 requiring further treatment should be treated with radiotherapy followed by PCV polychemotherapy. C: III; L: B.
- Patients with IDH-mutant and 1p/19q-codeleted oligodendrogliomas, WHO grade 3 should be treated with radiotherapy followed by PCV polychemotherapy (EORTC 26951, RTOG 9402)^79,80^. C: II; L: B.
- Temozolomide chemotherapy is standard treatment at progression after surgery and radiotherapy for most patients with IDH-mutant gliomas, WHO grade 2 or 3. C: II; L: B.

### IDH-wild-type glioblastoma, WHO grade 4

These tumours include histologic variants such as giant cell glioblastoma, gliosarcoma and epithelioid glioblastoma. Tumours formerly diagnosed as IDH-mutant glioblastoma are now referred to as IDH-mutant astrocytoma, WHO grade 4, and are managed either as IDH-wild-type glioblastoma or as IDH-mutant astrocytoma, WHO grade 3 (Table 2).

Surgery for glioblastoma should involve gross total resection whenever feasible^46^. A small RCT in patients aged >65 years at diagnosis of a WHO grade 3 or 4 glioma reported longer OS durations with resection versus biopsy^92^, but the relevance of this trial remains debatable owing to the limited sample size and KPS imbalances between treatment groups.

For decades, radiotherapy (60 Gy in 1.8–2 Gy fractions) has been the standard of care for glioblastoma, approximately doubling median OS durations^50^. Radiotherapy (50 Gy in 1.8 Gy fractions) improved OS relative to best supportive care in patients aged ≥70 years with a good KPS (≥70)^51^. Patients with unfavourable prognostic factors (defined by age and/or KPS) can be treated with hypofractionated radiotherapy (such as 40 Gy in 15 fractions), which has similar activity to irradiation with 60 Gy in 30 fractions^54^. Further hypofractionation to 5 × 5 Gy does not seem to compromise OS^93^ but is likely to cause neurocognitive adverse events if, in the future, elderly patients with glioblastoma live longer because of improved systemic treatment. Neither accelerated hyperfractionated or hypofractionated regimens nor brachytherapy, radiosurgery or a stereotactic radiotherapy boost are superior to standard radiotherapy regimens in terms of OS^57^. Concomitant radiotherapy and chemotherapy with temozolomide (75 mg/m^2^ daily throughout radiotherapy, including at weekends) plus six cycles of maintenance temozolomide (150–200 mg/m^2^, 5 out of 28 days) is the standard of care for adults with newly diagnosed glioblastoma who are in good general and neurological condition and are aged <70 years^59^. The addition of temozolomide to hypofractionated radiotherapy^54^ has also been shown to improve OS in patients aged ≥60 years^94^. The benefit from temozolomide is largely limited to patients with *MGMT* promoter-methylated glioblastoma^94,95^. The results of the NOA-08 (refs^96,97^) and Nordic trials^98^ led to *MGMT* promoter methylation testing becoming standard practice in many European countries for the management of elderly patients not considered eligible for combined modality treatment: patients with tumours lacking *MGMT* promoter methylation or of unknown *MGMT* promoter methylation status should be treated with hypofractionated radiotherapy alone whereas those with tumours with *MGMT* promoter methylation status should receive temozolomide alone (5 out of 28 days until disease progression or for 12 months)^97^. Until 2016, the broad consensus was that the results of all trials involving patients with tumours without *MGMT* promoter methylation showed no detriment from the omission of temozolomide^99^, challenging the view that this agent should be used in every patient regardless of *MGMT* promoter methylation status. This notion has become controversial again after a minor OS prolongation with radiotherapy plus temozolomide versus radiotherapy alone was observed in elderly patients with glioblastomas lacking *MGMT* promoter methylation^94^ and with the negative outcome of the CheckMate 498 trial^100^.

An open-label phase III trial of the addition of tumour-treating fields to maintenance temozolomide in patients with newly diagnosed glioblastoma revealed superior PFS and OS outcomes across all patient and tumour subgroups^101^, without relevant differences in QOL between arms^102^. However, questions have been raised regarding the mode of action, the study design without a sham control, the interpretation of data and the effect on health-related QOL in the general patient population^103^. Additionally, the feasibility and cost-effectiveness of tumour-treating fields as a standard of care for newly diagnosed glioblastoma remain highly controversial^104^. A focus on supportive and palliative care is appropriate for patients with large or multifocal lesions with a low KPS, notably if they are unable to provide consent for further therapy after biopsy^74^.

No benefit has been reported from increasing the dose of temozolomide in patients with newly diagnosed disease^105^ nor from extending the duration of chemotherapy beyond six cycles^106^. However, combining temozolomide with lomustine in the newly diagnosed setting might extend OS in patients with *MGMT* promoter-methylated glioblastoma^107^. This phase III trial involved a small cohort, did not show superior PFS for the combination and might deprive patients from lomustine, the standard of care at recurrence; thus, the use of this regimen appears to be largely restricted to some sites in German-speaking countries. The results of two phase III trials involving adults with glioblastoma demonstrated a prolongation of PFS (3–4 months) but not of OS when bevacizumab was added to temozolomide chemoradiotherapy^108,109^. A phase II trial involving a small cohort of elderly patients with *MGMT* promoter-unmethylated glioblastoma had similar results^110^; however, the clinical significance of such PFS gains is unclear because the reliability of assessing progression by neuroimaging can be questioned and because data from the RTOG 0825 trial raised concerns of early cognitive decline in patients treated with bevacizumab^109^. Bevacizumab has therefore not been approved for the treatment of newly diagnosed glioblastoma, with very few exceptions worldwide, but could be useful in patients with large tumours who are highly symptomatic and who might not otherwise tolerate radiotherapy. In the field of immunotherapy, negative phase III trials for OS include that of the EGFR-targeted vaccine rindopepimut in patients with EGFRvIII-positive glioblastoma^111^ and that of the immune checkpoint inhibitor nivolumab in patients with *MGMT* promoter unmethylated glioblastoma^100^.

**Fig. 3:**
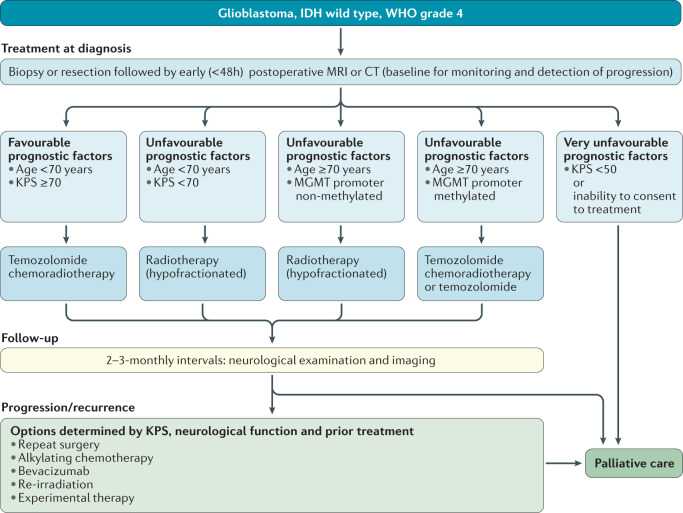
Clinical pathway for IDH-wild-type glioblastomas, WHO grade 4. KPS, Karnofsky performance status.

Standard-of-care treatments for patients with recurrent glioblastoma are not well defined; treatment is selected on the basis of prior therapy, age, KPS, *MGMT* promoter methylation status and patterns of disease progression (Fig. 3). Second surgery is an option for ~20–30% of patients, commonly with symptomatic but circumscribed relapses diagnosed not earlier than 6 months after initial surgery. Second surgery earlier than 6 months after initial surgery increases the risk of unnecessary intervention on the basis of pseudoprogression and is unlikely to provide durable benefit if the initial surgery followed by radiotherapy did not provide tumour control for more than a few months. Second surgery can also be considered upon early progression in symptomatic patients who might not have had adequate initial surgery. This procedure might improve post-recurrence survival in patients who are candidates for gross total resection of enhancing tumour^112^.

The efficacy of re-irradiation and the value of amino acid PET for target delineation remain debated. Radiation fractionation depends on tumour size. Larger lesions require smaller single fraction sizes to improve the safety and tolerability. Doses of conventional or near conventional fractionation have been tested as well as higher doses per fraction (5–6 Gy) using stereotactic hypofractionated radiotherapy to a total dose of 30–36 Gy or even radiosurgery with a single dose of 15–20 Gy, all with acceptable toxicity profiles^113^. The only RCT exploring bevacizumab plus radiotherapy versus bevacizumab alone reported improved PFS but not OS^114^.

The main systemic treatment options for patients with disease progression include nitrosoureas, temozolomide rechallenge, bevacizumab (depending on availability) or inclusion into a clinical trial. Lomustine (90–110 mg/m^2^) has never been shown to have superiority over another agent in an RCT^60^ but is increasingly considered as the most appropriate standard of care on the basis of its activity as the control arm of several RCTs^65,115^ and is also used in the AGILE trial^116^, with 6-month PFS rates of ~20%^60^. Similar results have been reported with alternative dosing schedules of temozolomide but activity is probably limited to patients with tumours with *MGMT* promoter methylation^117,118^. No data from RCTs support the view that dose-intensified schedules are superior to standard-dose temozolomide in patients with recurrent glioblastoma after a temozolomide-free interval.

Bevacizumab is not approved for patients with recurrent glioblastoma in the European Union, although it has been approved for this indication in other countries on the basis of objective response rates of ~30% in two uncontrolled phase II trials^66,67^. Bevacizumab has not been compared with placebo, and an effect on OS was not observed upon combination with lomustine as compared with lomustine alone^65^. To date, no active combination partner for bevacizumab to prolong OS has been identified. The main value of this agent in routine clinical practice is transient symptom control and the option for sparing treatment with steroids in symptomatic patients with large tumours.

In other studies of potential treatments for recurrent glioblastoma, nivolumab was not superior to bevacizumab^119^ and tumour-treating fields were not superior to physician’s choice of best treatment^120^. So far, only a limited role for targeted therapy in recurrent glioblastoma has been shown^12^. Approximately 50% of the rare epithelioid glioblastomas, an entity that remains controversial because of its similarity to anaplastic pleomorphic xanthoastrocytoma, harbour *BRAF*^V600E^ mutations. Patients with such tumours might benefit from BRAF inhibitors, at least in the setting of disease recurrence^121^.

### Recommendations

- The standard of care for patients with IDH-wild-type glioblastoma aged <70 years and with a KPS >70 includes resection as feasible or biopsy followed by involved-field radiotherapy and concomitant radiotherapy and six cycles of maintenance temozolomide chemotherapy (EORTC 26981-NCIC CE.3)^59^. C: I; L: A.
- Temozolomide might only be active in patients with *MGMT* promoter-methylated tumours whereas its activity in patients with *MGMT* promoter-unmethylated tumours is probably marginal^95^. C: II; L: B.
- Elderly patients not considered candidates for temozolomide chemoradiotherapy should be treated on the basis of *MGMT* promoter methylation status (NOA-08, Nordic Trial) with radiotherapy (such as 15 × 2.66 Gy) or temozolomide (5 out of 28 days) alone^96,98,97^. C: II; L: B.
- At recurrence, standards of care are less well defined. Surgery and radiotherapy might be considered. Nitrosourea regimens, temozolomide rechallenge and, with consideration of the country-specific label, bevacizumab are options of pharmacotherapy but an impact on OS remains unproven. When available, recruitment into appropriate clinical trials should be considered. C: II; L: B.

### H3 K27M-mutant diffuse midline glioma, WHO grade 4

This tumour type includes the majority of brainstem, thalamic and spinal diffuse gliomas in children and adults. Surgical options are limited and benefits from treatment options beyond radiotherapy have not been established because these tumours are rare and have not been studied in dedicated trials. In these tumours, the *MGMT* promoter is usually unmethylated. The prognosis of patients with this tumour type is poor.

### H3.3 G34-mutant diffuse hemispheric glioma, WHO grade 4

These tumours mostly occur in adolescents and young adults and the *MGMT* promoter is more often methylated than unmethylated^122^. These tumours were previously classified as IDH-wild-type glioblastomas and, thus, a reasonable treatment approach for such patients is chemoradiotherapy.

### Discouraged treatments

Steroids should not be given to treat asymptomatic or minimally symptomatic oedema and should be tapered as soon as possible, considering their unfavourable safety profile upon long-term administration. Furthermore, steroid use has been shown to be a negative prognostic factor for OS in patients with glioblastoma from three separate large cohorts^123^ and might interfere with the efficacy of radiotherapy, chemotherapy and immunotherapy.

Furthermore, we advocate against the use of any treatment beyond confirmed progression on that same treatment, including bevacizumab^124^ and tumour-treating fields, because the clinical benefit of this practice has not been established. Several chemotherapy regimens commonly used to treat other tumour types, including irinotecan and platinum compounds, are known not to be active against gliomas and should therefore not be used in this setting.

Given the poor outcomes of many patients with diffuse gliomas, new treatment concepts emerge and vanish that have never been tested in appropriate RCTs and the use of which outside a clinical trial is discouraged. Examples include the cocktail of repurposed drugs referred to as CUSP9, cannabinoids, methadone, sulfasalazine and valproate (except for seizure control).

## Conclusions

The revision of the WHO Classification of Tumors of the Central Nervous System^1^ has led to major changes in the way we routinely diagnose and treat patients with gliomas. The diagnosis and management plans should follow multidisciplinary tumour board recommendations throughout the course of disease. Multidisciplinary tumour board meetings are the fora for the discussion of whether treatments should be delivered locally or at a specialized centre and in inpatient or outpatient settings as well as to determine which neurorehabilitation measures would be useful. Local and national guidelines as well as EANO guidelines provide further guidance. Guidelines reflect knowledge and consensus at a given time; information on future updates will be posted on the EANO website. For many of the newly defined disease entities in the latest WHO classification, data on specific treatments and outcomes are not yet available; extrapolating data from clinical trials to these novel entities remains challenging. Well-designed, molecularly enriched RCTs are necessary to substantiate some of the treatment recommendations of the present guidelines.
