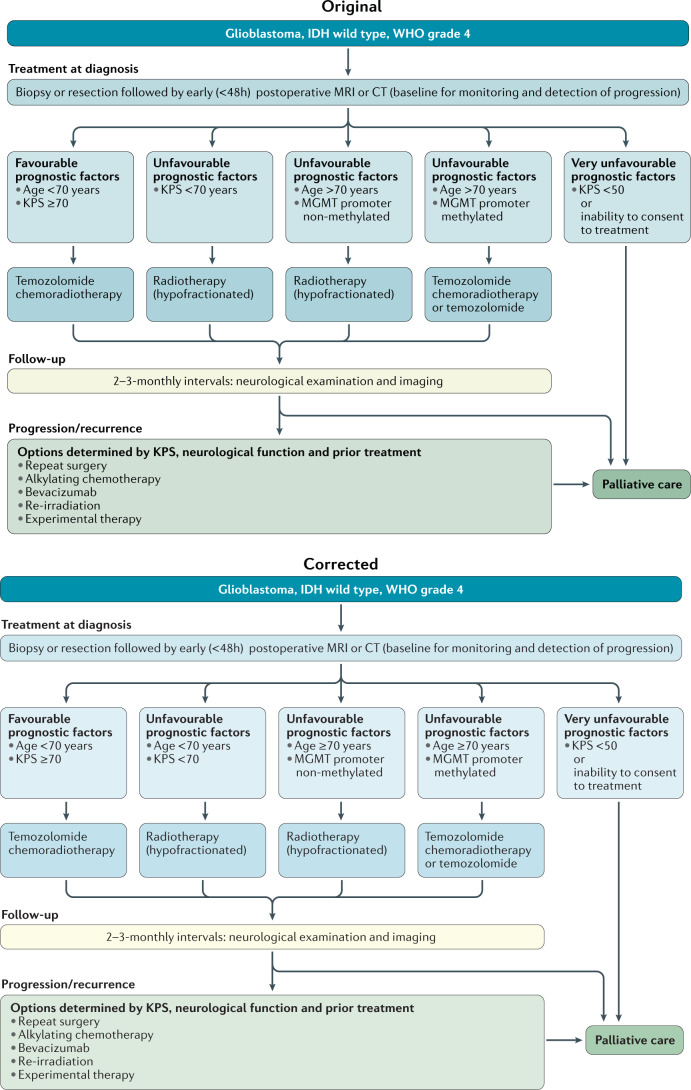# Author Correction: EANO guidelines on the diagnosis and treatment of diffuse gliomas of adulthood

**DOI:** 10.1038/s41571-022-00623-3

**Published:** 2022-03-23

**Authors:** Michael Weller, Martin van den Bent, Matthias Preusser, Emilie Le Rhun, Jörg C. Tonn, Giuseppe Minniti, Martin Bendszus, Carmen Balana, Olivier Chinot, Linda Dirven, Pim French, Monika E. Hegi, Asgeir S. Jakola, Michael Platten, Patrick Roth, Roberta Rudà, Susan Short, Marion Smits, Martin J. B. Taphoorn, Andreas von Deimling, Manfred Westphal, Riccardo Soffietti, Guido Reifenberger, Wolfgang Wick

**Affiliations:** 1 Department of Neurology, Clinical Neuroscience Center, University Hospital and University of Zurich, Zurich, Switzerland; 2 Brain Tumor Center at Erasmus MC Cancer Institute, University Medical Center Rotterdam, Rotterdam, Netherlands; 3 Division of Oncology, Department of Medicine I, Medical University of Vienna, Vienna, Austria; 4 Department of Neurosurgery, Clinical Neuroscience Center, University Hospital and University of Zurich, Zurich, Switzerland; 5 University of Lille, U1192 Lille, France; 6 Centre Hospitalier Universitaire (CHU) Lille, Neuro-Oncology, General and Stereotaxic Neurosurgery Service, Lille, France; 7 Oscar Lambret Center, Neurology, Lille, France; 8 Department of Neurosurgery, University Hospital Munich LMU, Munich, Germany; 9 Radiation Oncology Unit, Department of Medicine, Surgery and Neurosciences, University of Siena, Siena, Italy; 10 Department of Neuroradiology, University Hospital Heidelberg, Heidelberg, Germany; 11 Catalan Institute of Oncology (ICO), Hospital Germans Trias i Pujol, Badalona, Spain; 12 Aix-Marseille Université, Assistance Publique–Hôpitaux de Marseille (APHM), CHU Timone, Department of Neuro-Oncology, Marseille, France; 13 Department of Neurology, Leiden University Medical Center, Leiden, Netherlands; 14 Department of Neurology, Haaglanden Medical Center, The Hague, Netherlands; 15 Department of Neurology, Erasmus MC, Rotterdam, Netherlands; 16 Department of Clinical Neurosciences, University Hospital Lausanne, Lausanne, Switzerland; 17 Department of Neurosurgery, Sahlgrenska University Hospital, Gothenburg, Sweden; 18 Institute of Neuroscience and Physiology, Department of Clinical Neuroscience, Sahlgrenska Academy, Gothenburg, Sweden; 19 Department of Neurology, Medical Faculty Mannheim, Mannheim Center for Translational Neuroscience (MCTN), Heidelberg University, Mannheim, Germany; 20 German Consortium of Translational Cancer Research (DKTK), Clinical Cooperation Unit Neuroimmunology and Brain Tumor Immunology, German Cancer Research Center (DKFZ), Heidelberg, Germany; 21 Department of Neuro-Oncology, University Hospital, Turin, Italy; 22 Leeds Institute of Medical Research, St James’s University Hospital, Leeds, UK; 23 Department of Radiology and Nuclear Medicine, Erasmus MC, University Medical Center Rotterdam, Rotterdam, Netherlands; 24 Department for Neuropathology, University Hospital Heidelberg, Heidelberg, Germany; 25 DKTK and Clinical Cooperation Unit Neuropathology, DKFZ, Heidelberg, Germany; 26 Department of Neurosurgery, University Hospital Hamburg, Hamburg, Germany; 27 Department of Neuropathology, Heinrich Heine University Düsseldorf, Düsseldorf, Germany; 28 DKTK partner site Essen/Düsseldorf, Düsseldorf, Germany; 29 Neurology Clinic and National Center for Tumor Diseases, University Hospital Heidelberg, Heidelberg, Germany; 30 DKTK and Clinical Cooperation Unit Neurooncology, DKFZ, Heidelberg, Germany

**Keywords:** CNS cancer

Correction to: *Nature Reviews Clinical Oncology* 10.1038/s41571-020-00447-z, published online 8 December 2020.

In the original version of this Evidence-Based Guidelines, Fig. 3 contained errors regarding unfavourable factors for IDH-wild-type glioblastomas, WHO grade 4. The box label originally mentioning “KPS <70 years” has been corrected to “age <70 years; KPS <70 years”. In addition, greater clarity has been provided by changing “age >70 years” in other boxes to “age ≥70 years”. Fig. 3 has been corrected in the HTML and PDF versions of the article.

**Figure 1:**